# Social Medical Capital: How Patients and Caregivers Can Benefit From Online Social Interactions

**DOI:** 10.2196/16337

**Published:** 2020-07-28

**Authors:** Pietro Panzarasa, Christopher J Griffiths, Nishanth Sastry, Anna De Simoni

**Affiliations:** 1 School of Business and Management Queen Mary University of London London United Kingdom; 2 Centre for Primary Care and Public Health Barts and The London School of Medicine and Dentistry London United Kingdom; 3 Department of Computer Science University of Surrey Surrey United Kingdom

**Keywords:** online health communities, self-care, social networks, social capital, open and closed structures, social cohesion, brokerage

## Abstract

The rapid growth of online health communities and the increasing availability of relational data from social media provide invaluable opportunities for using network science and big data analytics to better understand how patients and caregivers can benefit from online conversations. Here, we outline a new network-based theory of social medical capital that will open up new avenues for conducting large-scale network studies of online health communities and devising effective policy interventions aimed at improving patients’ self-care and health.

Recent years have witnessed the rapid growth of online health communities targeted at patients with long-term conditions [1-3]. Patients and caregivers have increasingly used forums and social networks as alternative or complementary sources of support to more traditional forms of health care provision. Recent advances in network science and data-intensive analytics, combined with the growing advent of big data from social media, promise to yield new insights into how patients can tap the full potential of health communities to improve disease self-care and find the support and information they need [4,5].

Ongoing initiatives such as the Values in Action Champions [6], the Health Service Executive Quit Smoking Programme [7] and its Facebook community [8], the Public Health England Stoptober smoking cessation campaign [9], and NHS Digital [10] show how harnessing the power of social networks can help people to spread behaviors, affect cultural changes in the health service, and improve health and well-being outcomes. Similarly, our recent work on communities targeted at patients with respiratory conditions [11] and stroke survivors [3] has provided evidence regarding how certain patients can fare better than others simply by leveraging their communication patterns. What these studies suggest is the idea that some patients can be at an advantage simply because they are somehow better connected than others. In other words, a patient’s distinctive pattern of social engagement and communication in a community is an asset in its own right. That asset is what we call social medical capital.

As suggested by previous work on social capital and health care access, there is still controversy over the definition and measurement of social capital and its association with various types of health-related outcomes [12]. In this paper, we endorse a network-based perspective and describe a new conceptual framework for theorizing about the emergence of social medical capital as a function of communication patterns within online health communities. Previous studies of social capital in the health literature have also identified a variety of health-related outcomes that might be affected by social capital [12-15]. These include individuals’ health (eg, health status, mortality), health-related behavior (eg, health care–seeking behavior, illness self-management), access to local health services (eg, to community health clinics), and psychological well-being (eg, self-esteem, mutual respect). Here we focus only on two broad categories of outcomes (informational benefits and social support) and associate each of them with distinct network mechanisms viewed as structural sources of social capital.

We broadly define social medical capital as the advantages that any user (patient or caregiver) can gain from participation in the social networks provided by online health communities, where communication takes place at virtually no cost and across spatial and temporal boundaries. Like other forms of capital (eg, human or financial), social medical capital enables the achievement of certain ends (eg, emotional support); however, unlike other forms, it is based on the idea that social structure serves as a wellspring of advantages to users. That is, social medical capital is contingent on resources socially embedded in connections between users and accessible through these connections. This means not only that users can benefit from one another, but also that whether they can extract value from one another depends on how they interact [16,17]. This idea can be further articulated into four networking principles: (1) defragmentation, (2) bonding, (3) bridging, and (4) multiple-group membership.

## Defragmentation and Superusers

The value of participating in a community lies in the users’ ability to gain prompt access to a range of people. As more users join a community, a catalyst of social medical capital is structural defragmentation; that is, social interaction becomes more valuable as more pairs of users become mutually reachable along some path. Recent work has suggested that communities undergo defragmentation by self-organizing into hub-dominated structures [11]. The emergence of a small number of “superusers” (ie, the hubs engaging in conversations with a disproportionally large number of other users) engenders normative control and safeguards the communities from splitting into disjoint components [18].

## Bonding and Closed Structures

**Figure 1:**
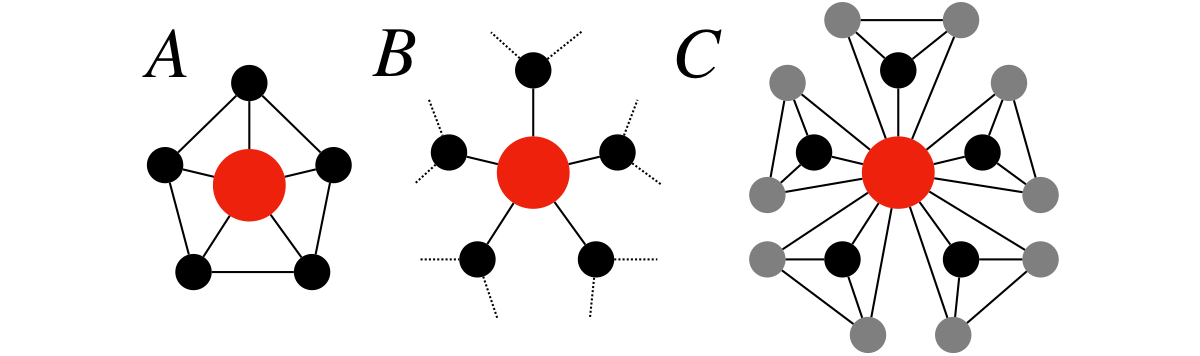
Three network structures associated with three distinct forms of social medical capital. The nodes represent the users, and each link refers to communication between users. The type of benefits that a focal user (the red node) can accrue from communication depends on whether the structure is: (A) closed, ie, the user’s partners also communicate with each other; (B) open, ie, the user acts as the intermediary between other users; or (C) mixed, ie, the user belongs to multiple densely connected groups and acts as the only intermediary between members of different groups.

In a socially cohesive closed network, links are forged locally, and pairs of connected individuals tend to be tied to at least one third party that they have in common (Figure 1A). Previous studies have suggested that closed structures, rich in third-party relationships, engender a shared identity and a sense of belonging, foster trust and cooperation, and sustain emotional support [16,17,19]. Evidence from two communities where patients primarily seek social support has shed light on the role of social cohesion in eliciting social medical capital [11]. Two empirical regularities have been uncovered: (1) highly connected support-giving superusers preferentially communicate with poorly connected support-seeking users; and (2) pairs of support-seeking users who communicate with the same support-giving superuser tend to communicate with each other as well, thus creating closed connected triangles centered on superusers. Local redundancy is therefore the structural engine of a support-oriented community.

## Bridging and Open Structures

An open network is rich in structural gaps and opportunities of intermediation between individuals (Figure 1B). Open structures, where connections tend to be weak and constitute “local bridges” between otherwise disconnected parts, have long been associated with informational benefits [16,17,20]. In an openly structured community, communication is likely to take users closer to complementary sources of information they do not already possess [21]. Brokerage opportunities between distinct social circles expose users to greater variance and novelty of resources, and eventually help them to satisfy their informational needs [20,21]. Paucity of local redundancy is therefore the structural engine of an information-oriented community.

## Multiple-Group Membership and Mixed Structures

In cases where the community aims to provide both emotional and informational support, social medical capital lies at the interface between bonding and bridging [17,22]. In such cases, the community will be most beneficial to the users if they can combine local redundancy with brokerage opportunities (Figure 1C). In a limiting case, this can be achieved when the following conditions are met: (1) a given user is a member of multiple clusters of users; (2) these clusters do not have any other users in common except the focal one (who therefore is the only intermediary between otherwise disconnected clusters); and (3) each cluster has an underlying closed cohesive structure (ie, all members are connected with one another).

## Research and Policy Roadmap

Empirically testing our theoretical framework will require the construction of large-scale longitudinal network data sets with information on users and their time-stamped messages. To comparatively assess the efficacy of network mechanisms of social capital, content analysis of messages would be needed so as to uncover how users’ phycological well-being and access to information vary as social connections also change over time.

To help policy makers to realize our vision of social medical capital as a crucial enabler of large-scale health care interventions in resource-constrained systems, we propose four targets: (1) promote patients’ and caregivers’ participation in online health communities to ensure continuous provision of socially embedded resources (eg, information, advice, support) that users can access through their (direct and indirect) social connections [14]; (2) support superusers’ role in online health communities through appropriate training programs [1]; (3) enhance the quality of peer support and patients’ self-care through robust evaluation systems; and (4) develop governance modes for maintaining privacy and confidentiality, cultivating trust and a participatory culture, and promptly detecting and preventing the spreading of misleading information and malicious behavior.
